# Oxidative Stability of Selected Edible Oils

**DOI:** 10.3390/molecules23071746

**Published:** 2018-07-17

**Authors:** Magdalena Maszewska, Anna Florowska, Elżbieta Dłużewska, Małgorzata Wroniak, Katarzyna Marciniak-Lukasiak, Anna Żbikowska

**Affiliations:** Division of Fats & Oils and Food Concentrates Technology, Department of Food Technology, Warsaw University of Life Sciences—SGGW (WULS-SGGW), 02-787 Warsaw, Poland; magdalena_maszewska@sggw.pl (M.M.); elzbieta_dluzewska@sggw.pl (E.D.); malgorzata_wroniak@sggw.pl (M.W.); katarzyna_marciniak_lukasiak@sggw.pl (K.M.-L.); anna_zbikowska@sggw.pl (A.Ż.)

**Keywords:** oxidative stability, edible oil, Rancimat test, Schaal Oven Test, storage

## Abstract

The aim of the study was to examine and compare oxidative stability of refined (peanut, corn, rice bran, grapeseed, and rapeseed) oils. The oils were subject a Schaal Oven Test (temperature 63 ± 1 °C) and a Rancimat test (temperature 120 °C) and their stability was compared at the 1st and 12th month of storage. Changes in the peroxide (PV) and anisidine (AnV) values in the thermostat test were the fastest in rapeseed oil and grapeseed oil. The best quality was preserved by peanut and corn oils both in the first and the twelfth month of storage. The induction times for the rice bran, corn, peanut, and rapeseed oils were similar from 4.77 h to 5.02 h in the first month and from 3.22 h to 3.77 h in the twelfth month. The shortest induction times were determined for grapeseed oil: 2.4 h and 1.6 h, respectively. A decrease of oxidative stability of about 30% was found in all the oils after 12 months of storage. The PV of 10, determined in the thermostat and Rancimat tests, were achieved at the latest in corn oil and the fastest in rice bran oil.

## 1. Introduction

Composition of fatty acids is particularly important in relation to oxidative stability of fat. The more unsaturated and less saturated a fat is, the faster the oxidation reaction proceeds [1,2]. Linolenic acid is oxidized the fastest, followed by linoleic and oleic acids [1]. That is why the fastest oxidation occurs in the grapeseed oil, which is characterized by the greatest amount of polyunsaturated fatty acids (about 68–85%), with the largest part constituted by linoleic acid (approx. 67%) [3,4]. Grapeseed, with a relatively high oil content (10–20 g/100 g seed), can also be considered as an oily raw material that is a rich source of natural antioxidants, such as vitamin E, polyphenols, and flavonoids [5,6,7]. Rapeseed oil, in comparison with other oils, contains the highest amount of linolenic acid (10–13%) [4,8]. In other oils, the percentage of this valuable acid is low (0–2%) [4,9,10,11,12]. In addition, it comprises about 20–26% linoleic acid in its composition [3]. The main advantage of this oil is a good ratio of n-6 to n-3 fatty acids (close to two to one), which meets optimal nutritional recommendations and a high C18:1 *cis* content, as well as a smaller amount of saturated fatty acids (SFA) (among popular vegetable fats) [13,14]. The peanut oil consists mainly of: Arachidonic, oleic, and stearic acids [3,15,16,17]. In addition, 21–35% of linoleic acid is present [15,16,17] and 0.1–0.4% of α-linolenic acid [15,16,18,19]. The high content of unsaturated fatty acids in peanut oil can be a problem because unsaturated fatty acids are susceptible to oxidation, which can cause changes in taste, aroma, and color [2]. Similarly, corn oil has a high nutritional value due to the high content of polyunsaturated fatty acids (PUFA), mainly linoleic acid (18:2) [19]. The acidic composition of corn oil is dominated by oleic acid (28%) and linoleic acid (50–58%) [4,17]. Furthermore, 0.4–2.0% of α-linolenic acid is present [4,8,17,20,21]. This oil is also rich in carotenoids and tocopherols. Carotenes have the function of provitamin A and tocopherols of vitamin E [22]. It is also one of the richest in plant sterols material. Rice bran oil attracts consumers’ interest thanks to high concentrations of health-promoting compounds, from tocopherols and tocotrienols, to phytosterols and γ-oryzanol [9]. In addition, it is one of the most nutritious edible oils due to its balanced profile of fatty acids [23]. The acid composition of rice bran oil is dominated by: Oleic (40–50%), linoleic (28–42%), and palmitic (16–21%) acids [24,25]. 

Oxidation stability is one of the most important quality parameters of edible vegetable oils. It determines their usefulness in technological processes as well as shelf life. In food chemistry, many methods are used to determine the oxidative stability of oils. The most reliable test is the storage test, but it takes too long. Therefore, methods that allow for determination of oil stability in the shortest possible time are valuable. The most frequently used include: Rancimat test, Schaal Oven Test—thermostatic test and chemical determinations of peroxide value (PV), anisidine value (AnV), and acid value (AV). The Rancimat test determines the oxidation stability of oils in a very short time, however, high temperatures (50–150 °C) and intensive aeration are used, which change the nature of the oxidation process. In the thermostat test, lower temperatures (30–63 °C) are applied without intense aeration, which results in fat oxidation time of up to several weeks, but the nature of changes is more similar to changes in natural storage conditions (storage test).

The aim of the study was to examine and compare oxidative stability of refined (peanut, corn, rice bran, grapeseed, and rapeseed) oils using a thermostatic test (temperature 63 ± 1 °C) and Rancimat test (temperature 120 °C) in the first and twelfth months of their storage. Chemical quality of the oils was also determined by determining the acid value (AV), the peroxide value (PV), the anisidine value (AnV), and the fatty acids composition.

## 2. Results

### 2.1. Fatty Acid Composition

**Figure 1:**
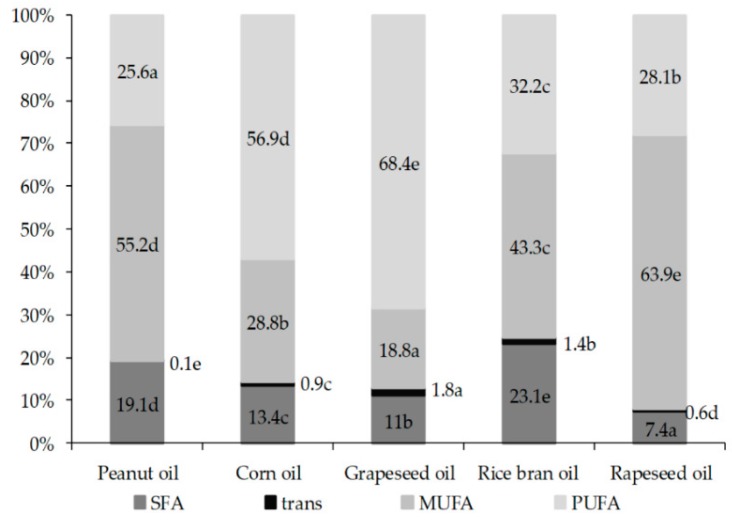
Fatty acid composition of the selected oils. Different small letters indicate statistically significant differences at the level *p* < 0.05 on columns for individual groups of fatty acids.

**Figure 2:**
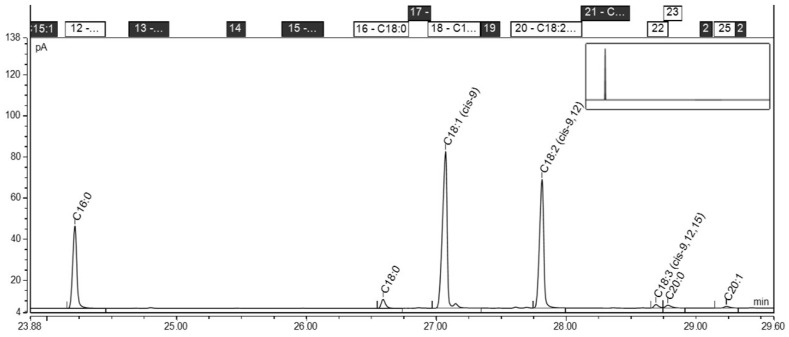
Chromatogram of rice bran oil.

Fatty acid composition of the studied oils is presented in Figure 1 and Figure 2. In the tested oils, unsaturated fatty acids were present in the *cis* configuration, while the *trans* isomers were in a small amount. Trace amounts of *trans* isomers (0.1%) were found in peanut oil, the largest amount (1.8%) contained grapeseed oil. The share of saturated fatty acids ranged from 7.4% in rapeseed oil to 23.1% in rice bran oil. The largest share of monounsaturated acids were found in rapeseed oil 63.9% and peanut oil 55.2%. Rice bran oil contained 43.3% of monoenic acids. Corn and grapeseed oils contained 28.8% and 18.8% of these acids, respectively. The largest share of polyene acids were found in grapeseed oil (68.4%), slightly lower in corn oil (56.9%). Peanut, rapeseed, and rice bran oils contained less than half polyene acids compared to grapeseed oil.

From a nutritional point of view, the content of polyene fatty acids in oils is a desirable feature. On the other hand, oils of this composition are not very resistant to external factors, they are susceptible to oxidation e.g., α-linolenic acid (18:3, ω-3) [26] or (punicic acid 18:3, ω-5) [27]. There is concern that long-term consumption of large amounts of linoleic acid might increase cancer risk [28,29].

The results for the fatty acids composition of the selected refined edible oils are similar to those obtained by other authors. Accordingly, peanut oil contains 19.1% of saturated acids, 55.2% of monounsaturated, and 25.6% of polyunsaturates [2,3,8,10,16,17,18,30]; in corn oil, there is 56.9% polyunsaturated fatty acids, 28.8% monounsaturated, and 13.4% saturated acids [3,4,8,17,20,21,31,32,33]. In rice bran, oil of saturated fatty acids accounts for about 23.1%, monounsaturated 43.3%, and polyunsaturated 32.2% [9,11,24,25]. Rapeseed oil, compared with other oils, contains the least saturated acids (7.4%) of about 63.9% of monounsaturated acids and 28.1% of polyunsaturates [3,4,8,13,33,34,35]; grapeseed oil contains a significant amount of polyunsaturated fatty acids—about 68.4%, 18.8% are monounsaturated, and 11% saturated fatty acids [3,4,5,8,9,26,36,37].

The changes in the acid, peroxide, and anisidine values determined during storage allow for the estimation of the quality of fat and establish its stability. Therefore, basic chemical quality determinations were carried out for fresh oils and after the twelfth month of storage. The results obtained are shown in Table 1 and compared with CODEX STAN 210-1999 [38].

The lowest values of peroxide and anisidine values in both the 1st and 12th months of storage were found for rapeseed oil, while the highest PV for rice bran oil, and AnV for grapeseed oil. PV and AnV values change slower over 12 months in rice bran oil, which indicates the slowest oxidation compared to the oxidation rate of the remaining samples tested. In the case of the acid value, the lowest initial value was found in peanut oil and the largest in grapeseed oil. After 12 months of storage, none of the tested oils exceeded the maximum permissible values of the acid, peroxide, and anisidine values. Roszkowska et al. (2015) [12] and Maszewska et al. (2018) [34] assayed in fresh rapeseed oils low AV 0.28 and 0.17 mg KOH/g and PV within 0.49–2.18 m Eq O_2_/kg. Hashemi et al. (2017) [7], for fresh grapeseed oil, marked PV equal to 0.7 m Eq O_2_/kg, while Pardo et al. (2009) [37] assayed PV, depending on the grape variety and extraction method, of between 5.99 and 13.50 m Eq O_2_/kg and AV between 0.37 and 1.47 mg KOH/g. Park et al. (2013) [39] assayed AV of fresh rice bran oil within 0.26–0.33 mg KOH/g, while Bakota et al. (2015) [11] assayed PV within 3.48–7.0 m Eq O_2_/kg and AnV between 7.2–23.0. Nepote et al. (2009) [40], Zhu et al. (2016) [30], and Idrus et al. (2017) [10] have assayed PV of fresh peanut oil within the limits 0.43–5.19 m Eq O_2_/kg, 0.37 m Eq O_2_/kg, and 1.05 m Eq O_2_/kg, respectively, AnV 0.14–1.09, 1.85, and 1.26, respectively. Zhu et al. (2016) [30] and Idrus et al. (2017) [10] assayed AV accordingly 0.09 mg KOH/g and 0.9 mg KOH/g. Baştürk et al. (2018) [41] assayed PV in refined corn oil equal to 9.96 m Eq O_2_/kg. TOTOX ranged from 1.7 to 13.6 in the analyzed oils (Table 1). Rice and grape oil had the highest values, which indicates a high degree of oxidation, despite the fact that they were in the initial period of shelf life. TOTOX below 10 is characteristic of fresh, high quality oils [42].

### 2.2. Schaal Oven Test

In fresh oils (1st month) and in oils opened after 12 months of storage, an accelerated Schaal Oven Test was carried out until PV equaled 100. Due to the various oxidative stability of oils, influenced by inter alia fatty acid composition, the duration of the test for individual oils was different.

Based on the data presented in Table 2, it was found that increase in peroxides varied for individual oils on subsequent days of thermostatting. The peroxide content increased slowly in all the tested oils in the early days of the test (2 days for grapeseed oil—up to 6 days for corn oil), the increase became more and more dynamic on the following days. Differences between peroxide values for particular oils were statistically significant. Rapeseed oil in the first days of the test oxidized the slowest. The rate of oxidative changes increased after the 5th day of thermostation. In the case of grapeseed oil, the lowest durability was found. A smaller difference in oxidative stability (determined in the 1st and 12th months of storage) of rice bran and grape oils was observed in comparison with rapeseed oil. The initial PV for peanut and corn oils was similar (no statistical differences), changes in peroxide content occurred gradually and slowly. In the case of peanut oil, the oxidation curve had the mildest course, but without a characteristic intense induction period. As a result, it was found that peanut oil underwent the slowest oxidation at 1 and 12 months of the storage, which shows its greatest stability. Similar changes in grapeseed oil were determined by Hashemi et al. (2017) [7]. Baştürk et al. (2018) [41] noted similar changes in corn oil, although they were slower.

**Figure 3:**
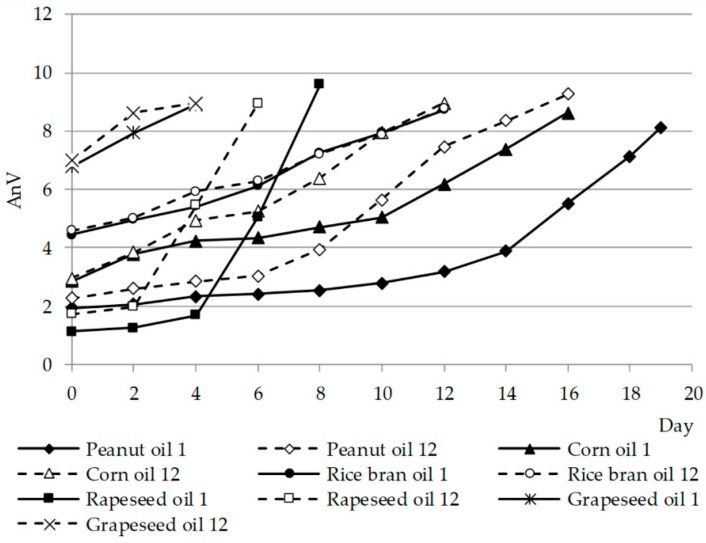
Anisidine (AnV) changes of the selected oils in the Schaal Oven Test in the 1st and 12th months of storage.

During the thermostat test, an anisidine value was determined at two-day intervals. Changes in the AnV value are shown in Figure 3.

It was observed that changes in the AnV value in grape, rice bran, peanut, and corn seed oils followed gradually and slowly on the subsequent days of thermostation both in fresh and stored oils. Peanut and corn oil oxidized slightly faster in the 12th month compared with the initial period. Only in rapeseed oil was there a sharp and distinct increase in the value of AnV, which proves that in rapeseed oil, secondary oxidation products are formed faster than in other oils. For grapeseed oil, an AnV = 8 was reached the fastest after just one day, however, this may be due to the fact that the initial AnV value in the oil was high (6.8 in fresh and 7 in stored oil), whereas this value was exceeded in peanut oil only after 18 days.

### 2.3. The Rancimat Test

**Figure 4:**
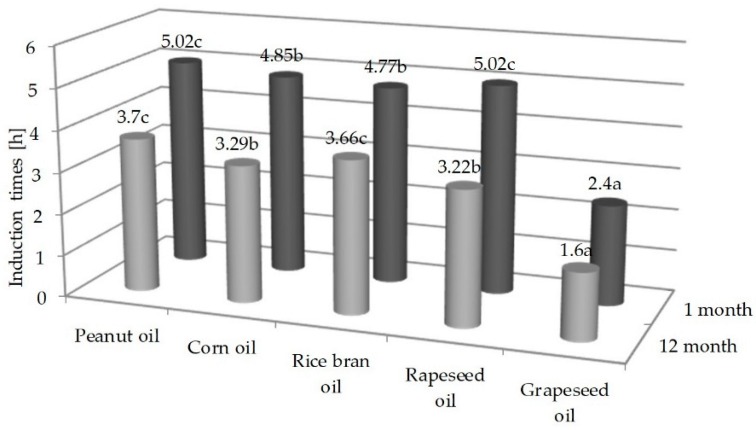
Induction time of the selected oils determined in the Rancimat test in the 1st and 12th months of storage. Different small letters indicate statistically significant differences at the level *p* < 0.05 between oils in month.

Conducting the Rancimat test allowed to determine the time of induction of the tested oils (Figure 4). Among the tested oils, the longest induction times in the first month of oil storage were determined for peanut oil and rapeseed oil (5 h), these oils were characterized by similar initial PV values (Table 1).

In the case of grapeseed oil, for which the initial PV value was 2.5 m Eq O_2_/kg, the induction time was about twice as short (2.4 h) as of the peanut oil. It was observed that the induction times for rice bran and corn oils were similar, 4.77 and 4.85 h, respectively. Rapeseed oil had the lowest and rice bran oil had the highest initial PV value among the tested oils (respectively, 0.26 and 4.51 m Eq O_2_/kg). It can therefore be concluded that there is no close relationship between the time of induction and the value of the initial PV. After twelve months of storage, the stability of oils measured by the time of induction decreased by 26%, 32%, 26%, 36%, and 33% for peanut, corn, rice bran, rapeseed, and grape oils, respectively. According to Roszkowska et al. (2015) [12], fresh rapeseed oils are characterized by a long induction time, i.e., about 10 h (at 110 °C), according to Maszewska et al. (2018) [34] and Redondo-Cuevas et al. (2018) [33], induction time for rapeseed oil at 120 °C is 4.3 h and 4.7 h. Pardo et al. (2009) [37] determined oxidative stability of between 6.38 h and 9.36 h in grapeseed oils at 98 °C and air flow 10 L/h. Redondo-Cuevas et al. (2018) [33] recorded the time of induction of corn oil equal to 0.84 h, Zhang et al. (2015) [31] (120 °C, air flow 10 L/h) put it at 3.51 h. According to Kowalski et al. (2004) [43] and Ratusz et al. (2016) [44], oils rich in polyene acids show convergent characteristics at higher temperatures in the Rancimat test.

**Figure 5:**
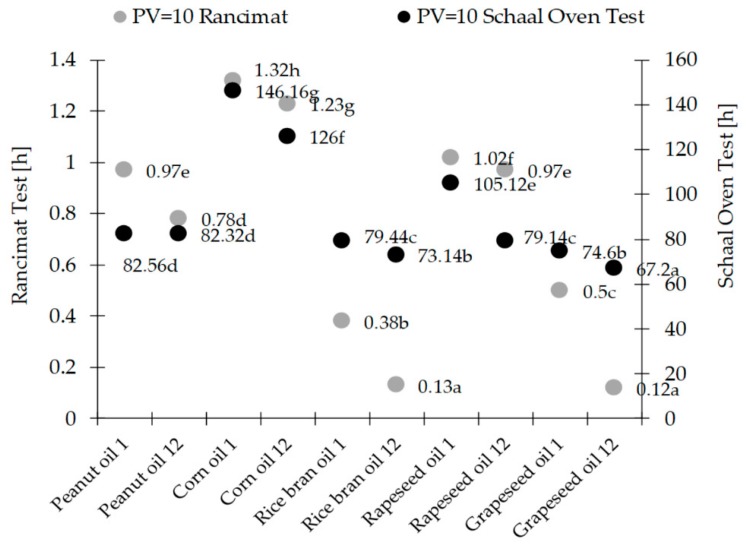
Time to reach peroxide (PV) = 10 in selected oils in the Rancimat and Schaal Oven Test in the 1st and 12th months of storage. Different small letters indicate statistically significant differences at the level *p* < 0.05 between oils in one test.

As part of the Rancimat test, the time at which PV reached the acceptable value of 10 m Eq O_2_/kg was determined. The rapeseed oil oxidation rate of change was significant and resulted from the presence of the highest amounts (approx. 68%) of polyunsaturated fatty acids. The rate of formation of the primary oxidation products grows as the amount of polyunsaturated fatty acids in oils rises. The fastest oxidation was observed for grapeseed oil, which contained about 68% polyunsaturated acids, the slowest changes were in peanut and rapeseed oils, which contained the smallest amounts of polyunsaturated acids (about 25–28%, respectively). Considering the results of the Rancimat test and the thermostat test, it was noted (Figure 5) that both in the 1st and 12th months of storage the longest time when the peroxide value reaches 10 was obtained for the corn oil. A slightly shorter time was noted for rapeseed and peanut oil. In the case of rice bran oil, a short PV = 10 time is due to the high initial oxidation state. Oxidative stability of oils is also influenced by antioxidants. The content of sterols in vegetable oils is from 70 to 1100 mg/100 g of oil. Most of these compounds are found in corn oil, then slightly less in rapeseed oil [45], γ-oryzanol in rice bran [39], and tocotrienols and tocopherols in grapeseed and rice bran oils [38].

The Rancimat and thermostatic tests were compared in the range of peroxide value up to 50 m Eq O_2_/kg of oil. The time at which selected peroxide values (PV = 10, 20, 30, 40, 50) were achieved in each method was read and correlation coefficients were determined (Table 2).

These values of correlation coefficients are very high (Table 3). This means that despite the use of different methods and different test conditions, changes in the tested oils were of similar nature and were comparable.

## 3. Materials and Methods

Selected refined oils (peanut, corn, rice bran, grapeseed, and rapeseed) purchased in stores were used for testing. The oils were selected which expired after about 12 months. The oils were stored in original light plastic bottles at room temperature (20 ± 2 °C), with light access (fixed oils on the shelf, without direct contact with the sunlight), in original packaging. The tests (Schaal Oven Test and Rancimat test) were carried out in the fresh (first month) and in oils opened after twelve months of storage. Oxidative stability of the oils was determined by Rancimat test [46] and Schaal Oven Test [47].

### 3.1. Chemical Analyses

Peroxide values (PV) of the oils were determined by iodometric technique (the results were shown in miliequivalent O_2_ per kg [m Eq O_2_/kg]) and acid value (AV) was determined by titration with 0.1 M ethanolic potassium hydroxide (the results were shown in mg KOH per gram [mg KOH/g]), while anisidine value (AnV) was determined in quartz cuvettes with the 10 mm optical path length on a Helios Gamma UV–Vis Spectrophotometer (USA) spectrophotometrically. The measurements were in accordance with the standard PV—ISO 3960:2017, AV—ISO 660:2009, and AnV—ISO 6885:2016 [48,49,50]. All the analyzed values were made in two samples in three replications.

### 3.2. Fatty Acid Analysis

The fatty acid composition of the oil samples was determined according to the AOCS Official Method Ce 1h-05, with minor modifications [51]. Fatty acid methyl esters (1 µL), prepared by ISO 5509:2000 standard method [52], were separated on a GC-FID system (TRACE™ 1300, Thermo Scientific, Waltham, MA, USA) equipped with a BPX 70 capillary column (length 60 m, i.d. 0.22 mm, film thickness 0.25 µm). Helium was used as a carrier gas at a flow rate of 1.5 mL/min. A split/splitless injector was operated at a temperature of 230 °C with a split rate set to 100:1, and the detector was the GC-FID. The GC’s oven temperature was programmed as follows: 80 °C hold for 2 min, ramped to 230 °C at a rate of 2.5 °C/min, hold for 5 min. Fatty acids were identified by comparing their retention times with authentic standards (RESTEK, Food Industry FAME Mix, catalog # 35077) and the results were reported as weight percentages.

### 3.3. Schaal Oven Test

Oil samples of 50 cm^3^ were stored in open beakers with a capacity of 100 cm^3^ in a thermostat at a temperature of ±63 °C. The retention time of the samples was dependent on the oil reaching a peroxide value of 100 m Eq O_2_/kg oil and an anisidine value of 8 [47].

### 3.4. Oxidative Stability by Rancimat Measurements

The oxidative stability was determined with 743 Rancimat apparatus from Metrohm, Herisau, Switzerland, according to ISO 6886:2016 [46], utilizing a sample of 2.50 g ± 0.01 g. All the samples were studied at the same temperatures of 120 °C under a constant air flow (20 L/h). The induction times [h] were printed automatically by the apparatus software with the accuracy of 0.005. During the Rancimat test, oil samples were taken and content of primary oxidation products (PV) generated during heating and aeration was tested. On the basis of the results, it was determined after what time the PV exceeded the value of 10 m Eq O_2_/kg oil, that is the maximum PV value presented in CODEX STAN 210-1999 [38] for refined oil used for consumption.

### 3.5. TOTOX

After determination of PV and p-AV, TOTOX values were calculated according to the formula TOTOX = 2PV + AnV.

### 3.6. Chemicals

All the solvents (chloroform, ethanol, methanol, and *n*-hexane) and reagents (acetic acid, potassium iodide, potassium hydroxide, sodium thiosulfate, starch soluble, and phenolphthalein) used were of analytical grade and purchased from P.O.Ch Co. (Gliwice, Poland).

### 3.7. Statistical Analysis

Statistical analysis was performed using Statgraphics 4.1 software. Data were expressed as Mean ± SD or as percentages. Variables were compared by Tukey Test, one-way Anova and the significance of differences among means was determined at *p* < 0.05. Different small letters with mean values in tables and diagrams indicate statistically significant differences. Statistical analysis was also used to determine the correlation coefficients when comparing the Schaal Oven Test and Rancimat tests. All the experiments were carried out in two samples in three replications (2 × 3).

## 4. Conclusions

The peanut and corn oils exhibited the best quality (AV, PV), both fresh and in the twelfth month of storage. In the thermostat test, the slowest changes in peroxide value occurred in corn oil. Changes in the peroxide and anisidine values in the thermostat test were the fastest in rapeseed and grapeseed oils. In the Rancimat test, the lowest oxidative stability (the shortest induction times 2.4 h fresh and 1.6 h storage oil) was characterized by grapeseed oil with the highest share of PUFA. The highest oxidative stability had rapeseed and peanut oils with the highest content of MUFA. The induction times for the rice bran, corn, peanut, and rapeseed oils were similar, from 4.77 h to 5.02 h in the first month and from 3.22 h to 3.77 h in the twelfth month. Oxidative stability of all oils after 12 months of storage decreased by about 30%. The thermostat test and Rancimat PV = 10 were determined in corn oil as the slowest and in rice bran oil the fastest (highest initial PV). The oxidative stability of the analyzed oils probably also depended on the different content of pro- and antioxidant compounds.

## Figures and Tables

**Table 1:** Changes in the acid (AV), peroxide (PV), and anisidine (AnV) values and TOTOX of the selected oils between the 1st and 12th months of storage.

Oil	AV (mg KOH/g)		PV (m Eq O_2_/kg)		AnV		TOTOX	
	1 Month	12 Month	1 Month	12 Month	1 Month	12 Month	1 Month	12 Month
Peanut	0.11a	0.17b	1.2a	1.6b	1.9a	2.3b	4.3a	5.5b
Corn	0.22a	0.25b	1.2a	1.7b	2.8a	2.9a	5.2a	6.3b
Rice bran	0.22a	0.34b	4.4a	4.5a	4.4a	4.6b	13.2a	13.6a
Rapeseed	0.17a	0.33b	0.3a	1.0b	1.1a	1.7b	1.7a	3.7b
Grapeseed	0.30a	0.39b	2.6a	3.0b	6.8a	7.0a	12a	13a
Codex Stan 210-1999 [37]	<0.6		<10		<8			

Different small letters indicate statistically significant differences at the level *p* < 0.05 on columns.

**Table 2:** PV changes of the selected oils in the Schaal Oven Test at 1st and 12th months of storage.

Days after Opening the Bottle	Peanut Oil		Corn Oil		Rice Bran Oil		Rapeseed Oil		Grapeseed Oil	
	1st	12th	1st	12th	1st	12th	1st	12th	1st	12th
0	1.18c	1.57d	1.24c	1.69e	4.51h	4.54h	0.26a	1.07b	2.63f	3.02g
1	2.76d	2.86de	2.55c	2.94e	6.41h	6.25h	1.91a	2.25b	3.66f	4.28g
2	5.49e	5.9f	3.4c	4.08d	8.03j	7.72i	2.19a	2.97b	6.53g	6.86h
3	8.64f	8.24e	4.06b	4.87c	9.62i	9.24g	2.72a	6.84d	9.41h	10.78j
4	11.79d	11.73d	4.72a	6.71c	15.57g	13.03e	5.86b	17.13i	14.69f	18.09h
5	16.38d	15.23c	6.94a	8.55b	21.65f	16.83e	16.67e	27.41i	24.02g	25.41h
6	20.97c	23.58d	9.18a	14.21b	27.88g	26.15f	25.85e	41.15j	36.44i	35.49h
7	28.08c	30.96d	17.64a	25.17b	35.24e	36.9f	38.83g	56.23j	49.25h	51.23i
8	30.2b	36.21c	26.74a	43.11e	42.92d	44.37f	50.68g	71.28j	63.7h	67.34i
9	35.36a	42.04b	42.2b	52.18e	49.86c	51.4d	62.79f	82.89i	75.02g	78.27h
10	44.85a	47.88b	48.32c	61.26e	57.06d	58.42d	75.26f	94.51h	91.2g	89.21g
11	45.34a	59.08b	58.92b	70.01d	66.11c	72.79e	88.59f	100.34h	103.16i	98.97g
12	53.95a	70.28b	69.55b	78.76c	82.37d	87.17e	103.63f	106.17g		108.72h
13	65.77a	77.71c	74.24b	86.59d	88.41e	91.95f				
14	70.76a	85.14c	78.57b	94.42d	94.47d	96.73e				
15	75.75a	92.58c	83.26b	102.25e	100.53d	101.5d				
16	82.42a	100.01c	94.28b							
17	88.58a		98.72b							
18	95.03a		107.09b							
19	101.56									

Different small letters indicate statistically significant differences at the level *p* < 0.05 on columns.

**Table 3:** Values of correlation coefficients between the Rancimat and Schaal Oven Test for selected oils.

Oil	Correlation Coefficients	
	1st Month	12th Month
Peanut	0.9957	0.9929
Corn	0.9727	0.9243
Rice bran	0.9994	0.9908
Rapeseed	0.9994	0.9892
Grapeseed	0.9832	0.9855
